# The Validation of a Novel Surveillance System for Monitoring Bloodstream Infections in the Calgary Zone

**DOI:** 10.1155/2016/2935870

**Published:** 2016-06-07

**Authors:** Jenine R. Leal, Daniel B. Gregson, Deirdre L. Church, Elizabeth A. Henderson, Terry Ross, Kevin B. Laupland

**Affiliations:** ^1^Department of Community Health Sciences, University of Calgary, 3280 Hospital Drive NW, TRW Building, Rm 3D10, Calgary, AB, Canada T2N 4Z6; ^2^Division of Microbiology, Calgary Laboratory Services, 9-3535 Research Road NW, Calgary, AB, Canada T2L 2K8; ^3^Department of Medicine, Health Sciences Centre, University of Calgary, Foothills Campus, 3330 Hospital Drive NW, Calgary, AB, Canada T2N 4N1; ^4^Department of Pathology and Laboratory Medicine, Diagnostic & Scientific Centre, 9-3535 Research Road NW, Calgary, AB, Canada T2L 2K8; ^5^Centre for Antimicrobial Resistance, University of Calgary, Alberta Health Services, Calgary Laboratory Services, 3330 Hospital Drive NW, B07 HMRB, Calgary, AB, Canada T2N 4N1

## Abstract

*Background*. Electronic surveillance systems (ESSs) that utilize existing information in databases are more efficient than conventional infection surveillance methods. The objective was to assess an ESS for bloodstream infections (BSIs) in the Calgary Zone for its agreement with traditional medical record review.* Methods*. The ESS was developed by linking related data from regional laboratory and hospital administrative databases and using set definitions for excluding contaminants and duplicate isolates. Infections were classified as hospital-acquired (HA), healthcare-associated community-onset (HCA), or community-acquired (CA). A random sample of patients from the ESS was then compared with independent medical record review.* Results*. Among the 308 patients selected for comparative review, the ESS identified 318 episodes of BSI of which 130 (40.9%) were CA, 98 (30.8%) were HCA, and 90 (28.3%) were HA. Medical record review identified 313 episodes of which 136 (43.4%) were CA, 97 (30.9%) were HCA, and 80 (25.6%) were HA. Episodes of BSI were concordant in 304 (97%) cases. Overall, there was 85.5% agreement between ESS and medical record review for the classification of where BSIs were acquired (kappa = 0.78, 95% Confidence Interval: 0.75–0.80).* Conclusion*. This novel ESS identified and classified BSIs with a high degree of accuracy. This system requires additional linkages with other related databases.

## 1. Introduction

Bloodstream infections (BSIs) constitute an important health problem and in severe cases have a high fatality rate [1]. Surveillance of BSIs is important to measure and monitor the burden of disease, evaluate risk factors for acquisition, monitor temporal trends in occurrence, and identify emerging and reemerging infections with changing severity. It is an area of growing interest because the incidence of antibiotic resistant bacteria is rising, and new resistant strains are emerging [2].

Traditional surveillance methods are dependent on manual collection of clinical data from the medical record, clinical laboratory, and pharmacy by trained infection control professionals (ICPs). This approach is time-consuming and costly and focuses infection control resources on counting rather than preventing infections [3]. Infectious diseases electronic surveillance systems are designed to obtain information from interrelated electronic databases in order to identify infection distributions within a particular setting [4]. Administrative and laboratory-based data may be linked for streamlined data collection on patient admission and demographic, diagnostic, and microbiologic information. With the increasing use and availability of electronic patient data within healthcare institutions and in community settings, ESSs may be developed and implemented with minimal cost and labour [4, 5].

As a result of uncertainty surrounding its accuracy, electronic surveillance has not been widely adopted. Traditionally, labour-intensive, manual infection surveillance methods remain the principal means of surveillance in many areas [5]. Consequently, there are few studies that have reported on the accuracy of electronic surveillance as compared to traditional manual methods.

The objective of this study was to evaluate previously developed, active, and electronic information, population-based surveillance system compared to traditional manual methods for bloodstream infections in the Calgary Zone [6].

## 2. Methods

### 2.1. Study Design

This study involved using a retrospective, population-based laboratory electronic surveillance system (ESS) [6]. The ESS database was developed by a multidisciplinary group of microbiologists, infectious disease specialists, and information technology and quality and safety experts. The ESS registered all residents of the Calgary Zone with a BSI since 2000. This database has been developed through linkages between regional microbiology and acute care hospital administrative databases. Specific algorithms were used to define incident episodes of BSI and allow their classification as either hospital-acquired (HA), healthcare-associated community-onset (HCA), or community-acquired (CA) [6].

### 2.2. Patient Population

The Calgary Zone (previously known as the Calgary Health Region prior to April 2009) is a fully integrated, publicly funded health system that provides virtually all acute medical and surgical care to the residents of the cities of Calgary and Airdrie and a large surrounding area including a number of smaller towns and communities (population ~1.2 million) in the province of Alberta. Calgary Laboratory Services (CLS) is a regional laboratory that receives all specimens submitted for blood culture testing from all three acute care hospitals and 24 community collection sites in the Calgary Zone [7, 8]. All adult patients (≥18 years of age) with positive blood cultures during 2007 were identified by CLS.

The ESS included a cohort of all patient types with a positive blood culture drawn at a site within the Calgary Zone: inpatient, outpatient, emergency, community, nursing home/long-term care, and out-of-region patients. Although patients managed in the community are included in the ESS, for the comparison study, only patients admitted to a hospital were included. Hospitalized patients were randomly selected from within the ESS cohort for detailed review and validation of revised electronic surveillance definitions based on the results by Leal et al. [6].

### 2.3. Comparison of the ESS with Medical Record Review

Data extracted from the ESS on the enrolled hospitalized patients included episodes of bloodstream infection, location of acquisition, and focal body source of the bloodstream infections. The ESS data was then compared for accuracy with similar data obtained by traditional medical record review (MRR). Chart reviews were done concurrently by a research assistant and an infectious diseases physician on all patients enrolled by the ESS using a standardized review form. All patient data were entered directly into a Microsoft Access 2003 (Microsoft Corp., Redmond, WA) database. The reviewers were unaware of the ESS classification of isolates, episodes of bloodstream infection, location of acquisition, and focal body source of bloodstream infections. The definitions used for the MRR and by the ESS are listed in Table 1 [9–13].

In both the ESS and MRR, patients with a BSI were considered to have hospital-acquired infections if they had been readmitted to a Calgary Zone hospital from a healthcare facility outside the region, where the length of hospital stay was unknown prior to transfer. Patients were considered to be residents of the Calgary Zone if they had a postal code or a residence that was listed within the 2003 boundaries of the region. Homeless patients treated in a Calgary institution and patients admitted to an Emergency Department within 1–3 months before blood culture collection were also considered to be residents if other location indicators were not available. Polymicrobial BSIs episodes were defined as a single incident infection caused by more than 1 type of bacteria/yeast within a 2-day period. This classification was determined by the integration of all clinical and microbiology data from the MRR or with the ESS.

### 2.4. Data Management and Analysis

Data were entered into Microsoft Access 2003 and analyzed using Stata 10.0 (StataCorp, College Station, TX). Random numbers were assigned to all ESS BSI episodes, and an* a priori* convenience sample of 300 patients was then randomly chosen for detailed review. An* a priori* convenience sample size of at least 300 episodes was used so that patients with BSI caused by a range of bacterial/yeast would be included within the practical study limitations. The number of incident episodes of BSI and the proportion of episodes that were HA, HCA, or CA infections in the ESS and the MRR were determined and compared descriptively. Concordant results were those in which the ESS and the MRR gave the same classification for BSI episodes, and discordant results were those in which the ESS and the MRR gave different classifications for BSI episodes. Whenever the MRR and the ESS gave discordant results, the BSI episode was further investigated. Agreement and kappa statistics were calculated using standard formulas and reported with binomial exact 95% Confidence Intervals (CIs). Bootstrap methods in the statistical software were used to determine 95% CIs because the classification of acquisition consisted of three categories. Kappa was used to measure the level of agreement between results from the ESS and the MRR for concordant BSI episodes for accurately establishing the location of acquisition and the source of infection. The kappa statistic was interpreted as follows: <0.20, poor, 0.21–0.40, fair, 0.41–0.60, moderate, 0.61–0.80, good, and 0.81–1.00, very good [14].

## 3. Results

### 3.1. The ESS

Overall, there were 1,492 incident episodes of BSIs among 1,400 adults in the Calgary Zone for an incidence rate of 156.1 per 100,000 population. The median age among adults with a recorded date of birth (99.8%) and one or more incident BSIs was 62.6 years (Interquartile Range (IQR): 48.4–77.7 years). The incident episodes of BSI occurred among 781 (55.8%) males. In 2007, there were 1,304 (93.1%) admissions to an acute care centre among patients with incident episodes of BSI. Of the 1,492 incident episodes of BSI, 360 (24%) were HA, 535 (35.9%) were HCA, and 597 (40.0%) were CA.

The 1,492 incident episodes of BSI were derived as follows. In 2007, there were 4,500 organisms isolated from blood cultures among adults. Most of the organisms (*n* = 3,530; 78.4%) were pathogenic organisms, while the rest (*n* = 970; 21.5%) were due to common skin contaminants. Of the pathogenic organisms, 1,834 (51.9%) were the first blood isolates within a 365-day window. Of these, 1,626 (88.7%) occurred in Calgary Zone residents. Twelve of these were subsequently excluded because they were unspeciated duplicates of pathogens isolated in the same blood culture. This left a total of 1,614 first blood isolates of pathogenic organisms in the blood cultures of which 1,383 (85.7%) and 109 (6.75%) were single isolate and polymicrobial incident BSIs, respectively.

### 3.2. Assessment of Agreement between the ESS and MRR

**Figure 1:**
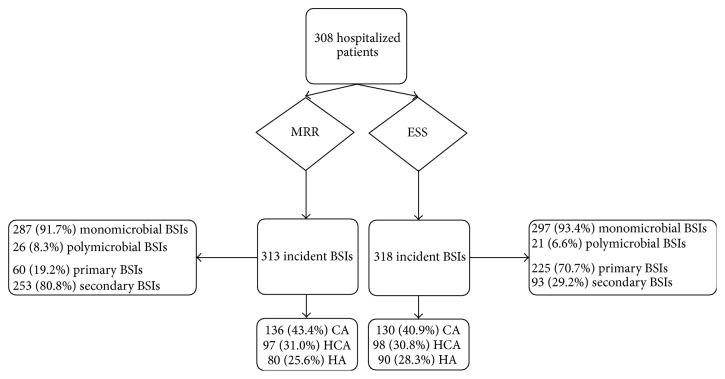
Distribution of incident bloodstream infections in the medical record review and the electronic surveillance system. HA: hospital-acquired; HCA: healthcare-associated community-onset; CA: community-acquired.

A total of 308/1,400 (22%) patients were randomly selected by the ESS and included in the analysis. A total of 661 blood cultures were drawn from these patients, and 693/4500 (15.4%) different pathogens were isolated. Overall, there were 329 episodes of BSIs in the 308 enrolled patients from the ESS. Among them, 313 were “true” BSIs, while 16 (4.9%) were classified as episodes of bloodstream contamination. The 313 BSIs occurred among 292 patients. The median age of these patients was 60.5 years (IQR: 48.6–75.9 years) and 158 (54.1%) were male.  Figure 1 outlines the number of incident episodes of BSI and a comparison of the ESS and MRR results obtained for their location of acquisition and the source of infection.

#### 3.2.1. Incident Episodes of BSI

MRR classified 313 (95%) episodes as true BSIs. The ESS classification for these patients was concordant in 304 (97%). Of the 9 discordant episodes identified by MRR, 4 were not identified in the ESS because patient had a previously positive blood culture with the same pathogen isolated in the prior 365 days. MRR therefore classified these patients as having a single episode of BSI. According to the MRR, another five patients had multiple BSI episodes (i.e., 2 or 3) but the ESS did not include all of them because the pathogens were not isolated for the first time in the prior 365 days. In comparison, the ESS identified 14 additional BSIs, two of which were not classified as separate episodes by the MRR. One of these was classified as polymicrobial by MRR, which the ESS classified as two separate single isolate BSIs due to the date of positive blood cultures and the fact that both pathogens were first blood isolates within the prior 365 days. In the other case, the MRR identified one BSI due to* Escherichia coli* which was considered to be contaminated with* Bacteroides fragilis*, but according to the ESS this was a separate BSI due to* B. fragilis*. The MRR erroneously classified* B. fragilis* as a contaminant in this case. The MRR classified all of the 12 other discordant BSIs identified by the ESS as bloodstream contaminants. Most of these were due to the collection of two blood cultures within 5 days of each other which grew coagulase-negative staphylococci (CoNS). The MRR classified these CoNS as contaminants but the ESS called them BSIs. Three episodes had only a single positive blood culture of* Rothia mucilaginosa*,* Lactobacillus*, and* Corynebacterium* species which were all classified as contaminants by the MRR.

#### 3.2.2. Acquisition Location of Episodes of Bloodstream Infection

All BSIs that were concordant between the ESS and the MRR (*n* = 304) were analyzed. Overall agreement was good between the ESS and MRR which was 85.5% in the patient's acquisition location for BSI, with an overall kappa of 0.78 (95% CI: 0.75–0.80).  Table 2 demonstrates the frequencies of the concordant and discordant episodes between the ESS and the MRR. The largest number of discrepancies occurred when the MRR classified BSIs as CA, while the ESS classified them as HCA (*n* = 19). These differences were attributed to missing information in the medical record on home care visits (*n* = 4), residency in a long-term care facility of nursing home (*n* = 3), and cancer therapy in a healthcare setting (*n* = 6). In five additional episodes, the ESS identified that the patients had previous hospitalizations, visits to the Home Parenteral Therapy Program (HPTP), or visits to the emergency room; however, these either were not identified by the MRR or were found to be related to the BSI identified during a subsequent hospitalization. The second largest group of discrepancies occurred when the MRR classified episodes of BSI as HCA, while the ESS classified them as CA (*n* = 15). Thirteen patients had one previous healthcare encounter identified by the reviewers, which the ESS did not identify. The healthcare encounters identified by the MRR included day procedures as outpatient prior to their BSI (*n* = 7), residents of home care (*n* = 3), transplant patients with multiple physician office visits (*n* = 2), and a previous hospital admission (*n* = 1). Two patients had two previous healthcare encounters identified (i.e., home care and/or previous hospital admission and resident of nursing home) by the MRR, which the ESS did not capture. Four BSI episodes were classified as HA by the ESS, which the MRR classified as HCA. Most (75%) of these discrepancies occurred because the ESS calculated that the blood cultures were obtained more than 48 hours after admission. In contrast, the MRR classified these BSIs as HCA because of the clinical assessment of the patient at admission and prior healthcare encounters. The MRR, however, did not have access to electronic timestamps like the ESS in order to accurately calculate time from admission to the positive blood culture. The ESS classified another four episodes of BSI as HA for the same reason, which the MRR classified as CA due to the date and time of blood culture collection being more than 48 hours from admission. Finally, two episodes were classified as HA by the MRR, and the ESS classified them as HCA. BSI occurred during hospital admission rather than being discharged from hospital prior to the BSI.

#### 3.2.3. Comparison of the Body Source of Infection between the Medical Record Review and the ESS

There was poor agreement (44.7%) between the ESS and the chart reviewers in classifying BSIs according to whether they were primary or secondary episodes with a low kappa score (*κ* = 0.11, 95% CI: 0.05–0.17). There were 168 (55.3%) discrepancies between the ESS and the MRR. The ESS classified most BSIs (161, 96%) as primary episodes which the MRR called secondary BSI episodes. This occurred because the ESS's laboratory database only contained blood culture results. According to the MRR, however, only 12 (7.5%) secondary BSIs had a positive culture or the same pathogen from another source which allowed confirmation of the MRR classification. All of the discrepancies (*n* = 7) classified by the ESS as secondary episodes of BSI but primary by MRR had a positive culture of the same pathogen from another body source. MRR showed that these BSIs were related to an intravenous device-associated infection.

## 4. Discussion

This study shows that the ESS is a valid tool for the accurate identification of incident episodes of BSIs. The ESS had a 97% concordance with MRR in identifying true episodes of BSI. The majority of discrepancies were due to multiple false positive blood cultures of CoNS being classified as true episodes of BSI by the ESS but as contaminants by the MRR. Blood cultures are susceptible to contamination at the time of collection, particularly by common skin contaminants such as CoNS and* Streptococcus viridans* group (SVG) [12]. It may be very difficult to sort out a “true” bacteremia from a false-positive blood culture when potential skin contaminants are recovered, without a detailed review of all available clinical, radiological, and laboratory data including all microbiology results. As previously described, inclusion of SVG as common skin contaminants in the development phase of the ESS resulted in an increased concordance of BSI episodes up to 95% [6]. Many of the SVG BSI episodes in this study were also clinically determined to be blood culture contaminants according to MRR. In this study, there was complete concordance between the ESS and the MRR for all “true” SVG BSIs.

The ESS also had a very good overall agreement of 85.5% in the classification of acquisition location of BSIs. Although this means that there still remains over 14% discrepancy, the ESS has the potential to be used as a screening mechanism for experienced infection control practitioners to quickly identify and investigate BSIs, especially HA BSIs, which are often the focus of infection prevention and control. The ESS classified a high number of BSI episodes as HCA, which the MRR classified as CA. A number of these were attributed to the ESS use of ICD-10-CA codes to identify patients with active cancer who were likely attending the regional cancer centre, which was not captured by the reviewers. During the development of the ESS it was recognized that BSI episodes in patients under active cancer treatment at the regional cancer centre would be misclassified as CA despite the cancer centre being located in one of the major hospitals. As a* post hoc* revision ICD-10-CA codes were added for active cancer to the ESS as a proxy for patients likely receiving cancer therapy. As previously described, this change significantly improved the ability of the ESS to accurately classify active cancer patients undergoing treatment as HCA [6]. The ESS performed better than MRR in accurately determining the acquisition of location of BSI for cancer patients who have significant exposure to various healthcare settings.

The current design of the ESS, however, had a low overall agreement (44.7%) with the MRR in accurately classifying BSI episodes as secondary to infection at another body source. The ESS as studied is therefore considered inaccurate for the application of assessing the foci of BSIs. This is attributed to the ESS database not currently containing sufficient clinical and radiological information to accurately assess whether a BSI is secondary to another source of infection. According to the MRR, 81% of true BSIs were classified as secondary, whereas the ESS classified only 29% this way. The identification of secondary BSIs by the MRR was mostly (66%) based on clinical information, physician diagnosis, or radiographic reports and not by a positive culture of the same pathogen at another body site. The identification of these infections by the ESS would be based solely on the recovery of pathogens from different infection sites, and in our randomly selected patients, only 12 had a positive culture for the same pathogen from another source. However, MRR does not always perform well either in accurately classifying BSIs in this regard. Despite a thorough clinical review, systematic studies show that the source of bacteremia or fungemia cannot be determined in one-quarter to one-third of patients [12, 13]. In addition, when the source of infection can be documented, only 25% are diagnosed by localized clinical findings, while another 32% were culture-proven. Further investigation is required to determine the optimal data sources or methodologies to improve and validate the classification of focal sources of BSI in our ESS. This limitation hinders the ESS's application in determining primary BSIs, particularly those due to device-associated infections, and reliably studying clinical severity and outcomes of primary versus secondary BSIs.

A number of other limitations of the ESS development merit discussion. Firstly, this study was retrospective and therefore the MRR was limited to clinical information that was previously documented. A prospective assessment may have led to some differences in the classification of episodes by MRR. Retrospective medical review is not frequently employed by ICPs in their identification of bloodstream and other infections, but rather they conduct prospective review of potential cases. By not conducting prospective review of medical records or by comparing the ESS to current infection prevention and control practices, this study is limited in describing the ESS's accuracy in conducting real-time or near-to-real-time surveillance. Despite this, the prospective evaluation of healthcare-associated infections by ICPs was shown to have large discrepancies and poor accuracy and consistency when compared with retrospective chart and laboratory review as the gold standard [15]. Secondly, this study only includes adults. The ESS already has the potential to identify all positive blood cultures among all residents in the Calgary Zone including children; however, validation and accuracy studies need to be conducted to ensure that episodes of BSIs and their location of acquisition are correctly classified in infants and children. Thirdly, MMRs were conducted concurrently by a trained research assistant and an infectious disease physician. Ideally, two or more teams or reviewers with an assessment of agreement between them would be preferred. Additionally, further assessments of interrater reliability between a trained medical record reviewer and an ICP would have been an adjunct to the evaluation of current surveillance methodologies employed by the region's infection prevention and control departments. Fourthly, the ESS only included data on BSIs and no other infections. There is potential to develop the ESS to evaluate other sources of infection determined by positive laboratory results. However, based on this analysis, the ESS did not perform well in classifying primary versus secondary BSIs when using laboratory data alone. Improvement in the identification of other infectious diseases may be accomplished by the introduction of automated pharmacy or prescription data and/or diagnosis codes from administrative data sources. Fifthly, there was no attempt to determine the rate of hospital-acquired device-associated BSIs or to determine qualitatively why they may have occurred, primarily due to the inaccuracy in classifying the focal source of the BSI. As part of the national and international emphasis on improving healthcare quality, rates of healthcare-associated infection have been proposed as quality measures for interhospital comparisons [16]. Central-venous catheter-associated BSI rates are a good measure of hospital's infection control practices, because these infections may be preventable [16].

In summary, surveillance data obtained with the ESS, which used existing data from regional databases, agreed closely with data obtained by MRR. Despite the limitations observed, the ESS has and can continue to have important implications for observational research, infection prevention and control, and healthcare quality improvement. The applicability of the ESS to other health systems is dependent on the types of databases in which information is stored, whether the data is readily accessible, the ability to link distinct databases into a relational database, and the quality of the data and the linkage. Because basic variables should be largely available to many other health systems, it is expected that the ESS can be applied in other healthcare jurisdictions.

## Figures and Tables

**Table 1:** Definitions for classifying bloodstream infections (BSIs) by the electronic surveillance system and by medical record review.

Classification	Definition		References
	Chart review	Electronic surveillance system	
Bloodstream infection	Patient has at least one sign or symptom, fever (>38°C), chills, or hypotension, and at least one of (1) pathogen recovered from >1 set of blood cultures and (2) isolation of organisms commonly associated with contamination^*∗*^ from >2 sets of blood cultures within 5 days	Pathogen recovered from >1 set of blood cultures or isolation of organisms commonly associated with contamination^*∗*^ from >2 sets of blood cultures within 5 days	[9]

Hospital-acquired	No evidence on the infection was present or incubating at the hospital admission, unless it was related to previous hospital admission	First positive culture obtained >48 hours after hospital admission or within 48 hours of discharge from hospital. If transferred from another institution then the duration of admission is calculated from admission time to first hospital	[9, 10]

Healthcare-associated community-onset	First positive culture obtained <48 hours of admission and at least one of (1) iv antibiotic therapy or specialized care at home other than oxygen, within the prior 30 days before bloodstream infection, (2) attending a hospital or hemodialysis clinic or iv chemotherapy within the prior 30 days before bloodstream infection, (3) admission to hospital for 2 or more days within the prior 90 days before bloodstream infection, and (4) resident of nursing home or long-term care facility	First positive culture obtained <48 hours of admission and at least one of (1) discharge from HPTP clinic within the prior 2–30 days before bloodstream infection, (2) attending a hospital clinic or ED within the prior 5–30 days before bloodstream infection, (3) admission to Calgary Zone acute care hospital for 2 or more days within the prior 90 days before bloodstream infection, (4) submission of a sample for culture from a patient who previously had a sample submitted from a nursing home or long-term care facility, (5) active dialysis, and (6) having an ICD-10-CA code for active, acute cancers as an indicator of those who likely attended or were admitted to the TBCC	[10–12]

Community-acquired	Bloodstream infections not fulfilling criteria for either hospital-acquired or healthcare-associated community-onset	First culture obtained <48 hours of admission and not fulfilling criteria for healthcare-associated community-onset	[10]

Primary BSI	Bloodstream infection is not related to infection at another site other than an infection associated with an intravascular device	No cultures obtained from any other site other than surveillance cultures or from intravascular devices within ±48 hours	[9, 13]

Secondary BSI	Bloodstream infection is related to infection at another body site (other than intravascular device) as determined on the basis of all available, clinical, radiographic, and laboratory evidence	At least one culture obtained from any other site other than surveillance cultures or from intravascular devices within ±48 hours	[9, 10]

^*∗*^Diphtheroids, *Bacillus *species, *Propionibacterium *species, coagulase-negative staphylococci, *Streptococcus viridans* group, and/or micrococci.

**Table 2:** Location of acquisition determined by the electronic surveillance system and the medical record review among concordant episodes of bloodstream infection.

Medical record review *n* (%)	Electronic surveillance system			Total *N* (%)
	*n* (%)			
	HA	HCA	CA	
HA	**77 (25.3)**	2 (0.7)	0 (0.0)	79 (26.0)
HCA	4 (1.3)	**72 (24.0)**	15 (4.9)	92 (30.3)
CA	4 (1.3)	19 (6.3)	**110 (36.2)**	133 (43.8)
Total *N* (%)	85 (28.0)	94 (30.9)	125 (41.1)	**304 (100.0)**

HA: hospital-acquired; HCA: healthcare-associated community-onset; CA: community-acquired.

## Additional Points

The objective of this study was to validate a previously developed electronic surveillance system (ESS) for bloodstream infections (BSIs) by assessing agreement with traditional medical record review (MRR). Infections were classified as hospital-acquired, healthcare-associated community-onset, or community-acquired. A random sample of 308 patients from the ESS was compared with independent MRR. Episodes of BSI were concordant in 304 (97%) cases. Overall, there was 85.5% agreement between ESS and MRR in classifying BSIs (kappa = 0.78, 95% Confidence Interval: 0.75–0.80).
